# Things We Do for No Reason – Ordering *Streptococcus Pneumoniae* Urinary Antigen in Patients With Community-Acquired Pneumonia

**DOI:** 10.1093/ofid/ofae089

**Published:** 2024-02-08

**Authors:** Matthew R Davis, Erin K McCreary, Alex M Trzebucki

**Affiliations:** Department of Clinical Pharmacy, Infectious Disease Connect, Inc., Pittsburgh, Pennsylvania, USA; Department of Clinical Pharmacy, Infectious Disease Connect, Inc., Pittsburgh, Pennsylvania, USA; Division of Infectious Diseases, Department of Medicine, University of Pittsburgh School of Medicine, Pittsburgh, Pennsylvania, USA; Division of Infectious Diseases, Department of Medicine, University of Pittsburgh School of Medicine, Pittsburgh, Pennsylvania, USA

To the  Editor— Community-acquired pneumonia (CAP) is 1 of the most common infectious diagnoses warranting hospitalization. Despite widespread vaccination, *Streptococcus pneumoniae* remains the most frequently implicated bacteria in CAP [1]. Consequently, guidelines recommend empiric and definitive antibiotic regimens with in vitro activity against *S pneumoniae* [2].

The diagnosis of CAP relies on clinical evaluation with consistent signs and symptoms such as cough, sputum production, pleuritic chest pain, fever, dyspnea, and hypoxia. Additionally, radiographic findings such as a focal infiltrate aid in confirming the diagnosis. However, microbiologic confirmation is often absent because it is challenging to obtain optimal respiratory samples. Indeed, no organism is identified in most CAP cases, requiring clinicians to treat empirically [1].

Rapid diagnostic and point-of-care tests are commonly used to circumvent the need for respiratory sampling and to expedite identification of a causative pathogen. Tests like the nasal influenza nucleic acid amplification test with high sensitivity and specificity are useful in confirming a diagnosis, expediting antiviral therapy, and avoiding empiric antibiotics. Therefore, use may be encouraged during periods of high seasonal prevalence. However, the *S pneumoniae* urinary antigen test (PUAT) is commonplace in the diagnostic workup of CAP despite its suboptimal sensitivity and the failure of a positive result to routinely impact clinical management [3].

We contend that PUATs should not be routinely included in the CAP diagnostic workup. First, the pretest probability of a positive test is low because most tests are negative (Table 1). Second, positive results do not alter antibiotic management in most cases because all recommended empiric antibiotic regimens for CAP have in vitro activity against *S pneumoniae* [2]. However, positive results do not exclude the presence of other pathogens, for which premature deescalation based purely on the PUAT result may lead to negative consequences, such as increased clinical relapse [10]. Although self-evident, negative PUAT results do not rule out the presence of other organisms either, and deescalation based on a negative PUAT is not necessarily warranted. Finally, a negative test does not definitively exclude the presence of *S pneumoniae* given suboptimal sensitivity of the test [3]. There is also evidence suggesting PUAT sensitivity is further decreased in the era of modern pneumococcal vaccination [11]. Additionally, it is unclear if sensitivity is further impacted by receipt of antecedent antibiotics, which is common for newly admitted patients with CAP [4, 13]. Notably, limited data exist evaluating PUATs in special populations of interest (eg, immunocompromised).

**Table 1.** Positivity Rate and Clinical Impact of *Streptococcus pneumoniae* Urinary Antigen

Reference	Tests Performed (n)^a^	Test Positivity (%)	Tests Affecting Antibiotic Therapy (%)^b^
Davis *et al.* (This article)	1428	1.4	0.07
Laijen *et al.* [12]	1150	14.5	6
West *et al.* [4]	1110	12	8.6
Engel *et al*. [5]	3479	6.2	2.1
Bellew *et al.* [6]	1941	4.2	Not specified
Dinh *et al.* [7]	979	5.2	0.7
Troy *et al*. [8]	170	7.6	2.9

^a^All tests ordered for any indication.

^b^Percentages of *Streptococcus pneumoniae* urinary antigen test ordered leading to any change in antibiotic therapy, which most reflected deescalation (ie, discontinuation of vancomycin, change from ceftriaxone to oral amoxicillin); tests were only considered clinically actionable when ordered for pneumonia indication.

Tests lack clinical utility and should not be used if the result will not impact clinical management, which is the case for PUATs where <9% of tests lead to changes in antimicrobial therapy [4–8, 12]. Given the prevalence of CAP as a presenting diagnosis, the widespread utilization of a diagnostic with low clinical utility becomes costly. These principles guided our recommendation to remove PUAT ordering at 1 of our tele-antimicrobial stewardship practice sites, a 3-hospital community health system with 565 total beds, resulting in projected annual laboratory test cost savings of $28 500. This decision was supported by a review of all PUAT orders in calendar year 2021, which comprised 1428 tests yielding 1408 (98.6%) negative results. Of the 20 (1.4%) positive tests, only 1 (0.07%) led to a change in therapeutic management.

Our experience is similar to that reported in the literature. In a large cohort study of patients admitted with pneumonia to 170 US hospitals from 2010 to 2015, 24,757 PUAT tests were performed in 159 894 admissions. Of these, 92.3% were negative and 97.7% failed to affect antibiotic selection [9]. A separate cohort study performed an economic assessment of the impact of 3479 PUAT tests at 2 hospitals found of all tests, 93.8% were negative or inappropriately ordered and 97.9% of tests failed to impact therapy leading to an excess cost per targeted treatment day of $319 (2012 $USD, not inflation-adjusted) [5].

It is important to consider special patient populations in which the risk-benefic calculation for PUATs may vary. Retrospective studies have observed a higher positivity rate for PUATs in severe pneumonia and in patients admitted to the intensive care unit; however, providers are less likely to deescalate antibiotics in this population, mitigating the benefit of optimized testing in this patient population [4, 7]. Additionally, regional variance of *S pneumonia* prevalence in CAP can impact PUAT performance characteristics such as test positivity, predictive value, and actionability. Notably, our test positivity, which included all PUATs performed in 2021, was lower than that of previous studies. Several of these only reported on PUAT performance in the subset of patients with an ultimate diagnosis of CAP. This likely inflates the clinical impact of PUAT results considering a substantial minority of PUATs (40%–44%) are performed on patients who do not have pneumonia, where PUATs provide no diagnostic utility [4, 5]. In West *et al.*, despite only assessing PUATs in patients with an International Classification of Diseases, 9th revision, code of pneumonia with radiographic confirmation, a minority of PUATs were positive (12%), with only 8.6% of total tests affecting management [4]. Considering CAP is typically 1 of several competing diagnoses on the differential, it is important to consider total test performance and not solely test performance after excluding non-CAP patients to understand its real-world clinical effectiveness.

Importantly, no benefit in clinical outcomes has been demonstrated for PUATs in randomized clinical trials, but concern for increased clinical relapse with targeted treatment based on PUAT testing has been raised as in the randomized clinical trial by Falguera *et al.* in which patients randomized to receive targeted treatment based on PUAT results had higher relapse rates (12% vs 3%, *P* = .04) [10]. As such, any purported benefit is attributed to the potential for PUATs to decrease time to deescalated antibiotic therapy, which is not sufficient to justify the costs of testing. Indeed, in the setting of updated IDSA guidelines for the treatment of community-acquired pneumonia, stewardship programs may focus on the initiation of targeted and even oral therapy at the beginning of the disease course rather than relying on PUAT to deescalate from unnecessarily broad empiric prescribing.

In conclusion, PUATs are common in the CAP diagnostic workup. However, the poor test characteristics and limited impact on antibiotic management renders them of low clinical utility. No benefit on clinical outcomes has been demonstrated in randomized trials, but potential harm has been identified. We believe that discontinuation of PUAT ordering is a safe, effective, and easily replicable means of practicing diagnostic stewardship, which could lead to reallocation of resources to more impactful testing or antimicrobial stewardship practices.

## Notes

***Patient consent statement***. This work did not include factors necessitating patient consent.

***Financial support***. We received no funding for this work.
